# Cross-national variation in the prevalence and correlates of current use of reusable menstrual materials: Analysis of 42 cross-sectional surveys in low-income, lower-middle-income, and upper-middle-income countries

**DOI:** 10.1371/journal.pone.0310451

**Published:** 2024-10-07

**Authors:** Nitai Roy, Md. Bony Amin, Md. Aktarujjaman, Ekhtear Hossain, Cyrus Mugo, Farhadul Islam, Mohammed A. Mamun, Manasi Kumar

**Affiliations:** 1 Department of Biochemistry and Food Analysis, Patuakhali Science and Technology University, Patuakhali, Bangladesh; 2 Faculty of Nutrition and Food Science, Patuakhali Science and Technology University, Patuakhali, Bangladesh; 3 Department of Biological Sciences and Chemistry, Southern University and A&M College, Baton Rouge, LA, United States of America; 4 Department of Global Health, University of Washington, Seattle, WA, United States of America; 5 Department of Research and Programs, Kenyatta National Hospital, Nairobi, Kenya; 6 Department of Biochemistry and Molecular Biology, University of Rajshahi, Rajshahi, Bangladesh; 7 CHINTA Research Bangladesh, Dhaka, Bangladesh; 8 Department of Public Health and Informatics, Jahangirnagar University, Dhaka, Bangladesh; 9 Department of Public Health, University of South Asia, Dhaka, Bangladesh; 10 Institute for Excellence in Health Equity, New York University School of Medicine, New York, NY, United States of America; Cranfield University, UNITED KINGDOM OF GREAT BRITAIN AND NORTHERN IRELAND

## Abstract

**Objectives:**

This study investigates the prevalence of the use of reusable menstrual materials in LMICs, examines differences in prevalence between countries and areas, and identifies individual and country-level factors associated with their use.

**Methods:**

Data from Multiple Indicator Cluster surveys conducted between 2017 and 2020 in LMICs were used. Prevalence estimates and 95% CIs were calculated for overall, rural, and urban areas. Multivariable logistic regression was used to identify individual and country-level factors associated with the use of reusable menstrual materials.

**Results:**

The study included 42 surveys from LMICs, with 1653850 weighted women and girls aged 15–49 years. The overall prevalence of the use of reusable menstrual materials was 12.1% (95% CI 12.1–12.2), with significant variation between and within countries, ranging from 0.5% (0.3–0.8) in Serbia to 97.2% (96.5–97.9) in Sao Tome and Principe. The prevalence was higher in rural areas (23.9% [23.8–24.0]) than in urban areas (6.2% [6.2–6.2]), with significant differences between most countries. Use of reusable menstrual materials was associated with lower education levels, being married, low economic status, living in Asia and Africa, living in countries with lower GDP, living in rural areas, and limited availability of private places to wash menstrual materials. The prevalence of the use of reusable menstrual materials had an inverse linear relationship with the country’s GDP.

**Conclusions:**

The study found that the use of reusable menstrual materials is more prevalent among women and girls in rural areas, those with lower education levels, lower economic status, and those living in countries with lower GDP. Given these disparities, policies and initiatives targeted at improving menstrual health in LMICs should focus on socioeconomically disadvantaged groups to ensure they have access to safe and appropriate menstrual materials.

## 1. Introduction

“Menstrual health is a state of complete physical, mental, and social well-being and not merely the absence of disease or infirmity, in relation to the menstrual cycle” [1]. The World Health organization (WHO) and United Nations International Children’s Emergency Fund (UNICEF) Joint Monitoring Programme (JMP) emphasize the need for “clean menstrual management material to absorb or collect menstrual blood, that can be changed in privacy as often as necessary, using soap and water for washing the body as required, and having access to safe and convenient facilities to dispose of used menstrual management materials” [2]. The broader concept of menstrual health encompasses knowledge of self-care and the menstrual cycle, access to menstrual care services, the ability to make informed decisions about personal care, diagnosis and treatment of any menstrual-related discomfort, and a respectful environment for this natural process [3].

Ensuring optimal menstrual hygiene management is critical to sexual and reproductive health and is a fundamental human right [4]. This right entails the provision of clean materials for managing the menstrual flow and facilities for washing and disposing of used products, as well as privacy and the ability to change products as needed during the menstruation period [5]. UN Women also emphasizes the importance of sanitation that respects human dignity and caters to the unique needs of women and girls [6]. Access to proper sanitation facilities and menstrual hygiene management resources can significantly impact the physical and mental well-being of menstruators, particularly in low-income settings. Therefore, menstrual hygiene management must be prioritized to ensure that everyone has access to the resources they need to manage their menstruation safely and with dignity [7].

Unfortunately, out of the 1.9 billion menstruating women and girls, around 500 million do not have adequate facilities for menstrual hygiene management worldwide [8]. In low- and middle-income countries (LMICs), inadequate access to safe and clean water, sanitation facilities, hand hygiene facilities, puberty education, and inappropriate waste disposal systems exacerbate public health challenges [9]. This results in limited menstrual hygiene management and restricted access to hygiene materials, further reducing the quality of life for menstruating individuals and placing a substantial burden on the healthcare sector [10].

There are two main types of menstrual materials: single-use materials (including tampons and disposable sanitary pads) and reusable materials (including cloth, washable sanitary pads, and menstrual cups) [11]. Women and girls’ preferences for menstrual products vary depending on personal preference, economic status, cultural beliefs, and local market availability [12]. While it is likely that moist reusable pads may increase reproductive tract infections (RTIs) risk, reusable menstrual cups do not have these same challenges and have been shown to reduce RTIs risk overall [13, 14]. The wide variety of improvised materials encompasses a range of options, extending from reused cloth such as old clothes and rags to clean newly acquired clothes. The utilization of old clothes among women has the potential to increase susceptibility to infections and provoke allergic reactions in the sensitive skin surrounding the genital area [15]. However, women and girls in LMICs face difficulties washing and changing reusable menstrual materials, which increases the risk of RTIs [16, 17]. Adequate menstrual product options can enable girls and women to continue with their daily activities, work, or education without the fear of menstrual blood leakage [18].

Girls and women face various obstacles when attempting to find the most suitable menstrual products. These obstacles include lack of information, prejudice, cultural norms, lack of means, location, safety fears, and limited availability [18]. The evidence on the use of reusable menstrual materials is varied. Some studies suggesting that reuse may be associated with urinary tract infections (UTIs) and poor hygiene-related health conditions [16]. However, other studies argue that, when used appropriately, reusable products can be both environmentally sustainable and as effective as any other product [12, 19]. Emerging research is also exploring the effectiveness of menstrual cups in comparison to other menstrual products, with some findings suggesting that they may offer certain advantages in reducing the risk of reproductive tract infections (RTIs) [18]. However, more research is needed to draw definitive conclusions.

The JMP Progress reports, such as the one titled "Progress on household drinking water, sanitation, and hygiene 2000–2020: Five years into the SDGs," have provided an evaluation of the utilization of reusable menstrual materials on a country-specific basis [20]. However, the existing body of empirical research on the correlates of utilizing reusable menstrual materials, particularly in relation to sociodemographic and socioeconomic factors, remains limited. Additionally, the association between the use of such materials and a country’s Gross Domestic Product (GDP) lacks substantial empirical evidence. Therefore, this research endeavor aimed to evaluate the extent to which reusable menstrual materials are utilized in LMICs, while also identifying individual and country-level factors that may be linked to the use of reusable menstrual materials.

## 2. Methods

### 2.1. Background, data source, and data quality

The Multiple Indicator Cluster Surveys (MICS) program is a household survey program financed by UNICEF with the main goal of monitoring the situation of women and children. Midway through the 1990s, MICS was developed, giving nations access to vital survey instruments (such as questionnaires, an implementation manual, and sample instructions). To increase survey capacity, MICS has evolved into a full-fledged survey program that offers a comprehensive set of tools and technical support for all phases of implementation at the national, regional, and international levels. MICS runs multiyear rounds and often introduces new tools at the beginning of each round. The sixth and largest cycle of surveys for the program (MICS7) is currently underway, with the majority of them taking place in West and Central Africa and Europe and Central Asia.This study is a cross-sectional analysis that utilizes publicly available data from the (MICS) (https://mics.unicef.org/surveys).

MICS are nationally representative surveys, conducted mostly in LMICs, that provide information on various aspects of households, as well as information related to reproductive health, hygiene practices, health status, and others. The surveys employ a multi-stage cluster sampling technique to select women and girls aged 15–49 years. Each dataset includes a previously calibrated frequency weight and is applicable within the country. According to the instructions, we applied the provided frequency weight to the specified dataset to address the issues of over- and under-sampling across different strata and account for variations in nonresponse. Incorporating data quality assurance measures into individual surveys is an essential component of the survey process. As an integral component of survey findings reports, countries generate a comprehensive compilation of data-quality tables [21]. These tables encompass a diverse array of essential indicators, thereby offering users valuable insights into the survey’s performance.

### 2.2. Selection of surveys, dependent and independent features

For this study, the most recent MICS survey, MICS 6, was utilized. The dependent variable in this particular study pertaining to the utilization of reusable menstrual management materials. The study referred to the utilization of materials during the previous menstrual cycle (were the materials reusable?) [11]. This item was initially assigned the values Yes = 1, No = 2, and Don’t Know = 8 for its coding. The results were recoded as "Yes" equaling 1 and "No" equaling 0 before the analysis was performed. Responses that indicated either ’don’t know’ or ’non-response’ were removed from the dataset. Independent features, such as age, education, union status (marital status), wealth index quantile, area, region, and availability of a private place for washing, were also considered [11, 22]. We examined earlier surveys for the same dependent and independent features, but we excluded MICS 3 and 5 because they did not collect data on the use of reusable menstrual materials. We then downloaded all available MICS 6 datasets and searched for the retrieval of the same information. We excluded surveys that lacked one or more of the dependent or independent variables, resulting in 42 surveys (38 LMICs) (some countries had more than one survey conducted as part of the MICS to capture regional disparities within their populations). We obtained GDP per capita and the survey country’s economy from the World Bank’s website and evaluated them as independent variables.

### 2.3. Sample information

Our study includes 42 surveys, with a total of 471364 responses to the " use of reusable menstrual materials" question. We excluded 1036 responses due to missing values, missing weights, no response, or unknown response. Our final sample size was 470328, which we weighted using MICS-supplied weights, resulting in a sample size of 1653850 (rural = 554869, urban = 1098981) (The MICS surveys never define the urban-rural in a surveyed country. The urban-rural definition is country-specific; they may vary from country to country. Some are population size based, some are infrastructure based); **Table 1** provides further details.

**Table 1:** Sample characteristics.

Sl. No	Surveys	Region	Country’s economy	GDP (US $)	Survey year	Response on "Menstrual materials reuse"	Sample excluded (Missing values, don’t know, No response)	Unweighted sample size	Weighted sample size	Urban	Rural
*1*	Central African Republic	West & Central Africa	Low income	476	2018–2019	6795	27	6768	6718	2624	4094
*2*	Chad	West & Central Africa	Low income	685	2019	17094	27	17067	17328	3631	13697
*3*	Democratic Republic of the Congo	West & Central Africa	Low income	560	2017–2018	15169	19	15150	16033	8582	7451
*4*	Guinea Bissau	West & Central Africa	Low income	803	2018–2019	815	2	813	850	409	441
*5*	Sierra Leone	West & Central Africa	Low income	496	2017	13077	17	13060	13286	6729	6557
*6*	The Gambia	West & Central Africa	Low income	732	2018	11784	13	11771	11938	8632	3305
*7*	Togo	West & Central Africa	Low income	830	2017	5816	4	5812	5855	2970	2885
*8*	Madagascar	Eastern & Southern Africa	Low income	523	2018	13003	11	12992	13178	3813	9365
*9*	Malawi	Eastern & Southern Africa	Low income	636	2019–2020	20028	5	20023	19950	3909	16041
*10*	Bangladesh	South Asia	Lower middle income	1855	2019	55824	90	55734	56059	13420	42639
*11*	Nepal	South Asia	Lower middle income	1194	2019	12486	2	12484	12625	8859	3766
*12*	Pakistan Punjab	South Asia	Lower middle income	1482	2017–2018	61129	47	61082	60859	23794	37065
*13*	Pakistan Sindh	South Asia	Lower middle income	1288	2018–2019	22099	136	21963	22173	13177	8995
*14*	Kiribati	East Asia & Pacific	Lower middle income	1695	2018–2019	3440	6	3434	3446	2040	1406
*15*	Lao PDR	East Asia & Pacific	Lower middle income	2455	2017	17946	32	17914	18251	7427	10824
*16*	Mongolia	East Asia & Pacific	Lower middle income	4156	2018	8850	43	8807	8623	6009	2614
*17*	Samoa	East Asia & Pacific	Lower middle income	4067	2019–2020	3544	26	3518	3515	742	2773
*18*	Kyrgyz Republic	Europe & Central Asia	Lower middle income	1308	2018	5006	11	4995	5005	2020	2985
*19*	Ghana	West & Central Africa	Lower middle income	2275	2017–2018	12524	4	12520	12582	6508	6073
*20*	Sao Tome and Principe	West & Central Africa	Lower middle income	1987	2019	2497	11	2486	2431	1584	847
*21*	Lesotho	Eastern & Southern Africa	Lower middle income	1192	2018	5458	8	5450	5530	2689	2841
*22*	Zimbabwe	Eastern & Southern Africa	Lower middle income	1316	2019	8368	1	8367	8355	3440	4915
*23*	Algeria	Middle East & North Africa	Lower middle income	4142	2017–2018	31381	97	31284	31454	20056	11398
*24*	State of Palestine	Middle East & North Africa	Lower middle income	3239	2019–2020	6145	39	6106	6176	4798	1378
*25*	Tunisia	Middle East & North Africa	Lower middle income	3680	2018	5320	9	5311	5431	3763	1669
*26*	Honduras	Latin America & Caribbean	Lower middle income	2567	2019	17119	61	17058	17172	8486	8686
*27*	Tuvalu	East Asia & Pacific	Upper middle income	4143	2019–2020	691	2	689	690	478	212
*28*	Tonga	East Asia & Pacific	Upper middle income	4903	2019	2553	12	2541	2504	580	1924
*29*	Kosovo (Roma settlements)	Europe & Central Asia	Upper middle income	4416	2019–2020	1387	5	1382	1383	748	635
*30*	Kosovo	Europe & Central Asia	Upper middle income	4416	2019–2020	4981	8	4973	4970	2110	2860
*31*	Montenegro	Europe & Central Asia	Upper middle income	8845	2018	2077	4	2073	2089	1433	656
*32*	Republic of North Macedonia (Roma Settlements)	Europe & Central Asia	Upper middle income	6070	2018–2019	1308	2	1306	1304	1215	89
*33*	Republic of North Macedonia	Europe & Central Asia	Upper middle income	6070	2018–2019	2964	2	2962	2976	1896	1080
*34*	Serbia (Roma Settlements)	Europe & Central Asia	Upper middle income	7417	2019	1651	2	1649	1662	1134	528
*35*	Serbia	Europe & Central Asia	Upper middle income	7417	2019	3501	1	3500	3468	2181	1287
*36*	Turkmenistan	Europe & Central Asia	Upper middle income	7612	2019	4910	0	4910	4902	2215	2687
*37*	Iraq	Middle East & North Africa	Upper middle income	5523	2018	16658	9	16649	16106	11316	4790
*38*	Costa Rica	Latin America & Caribbean	Upper middle income	12469	2018	6610	23	6587	1187627	876986	310641
*39*	Dominican Republic	Latin America & Caribbean	Upper middle income	8282	2019	20217	66	20151	20264	15584	4680
*40*	Cuba	Latin America & Caribbean	Upper middle income	9099	2019	8126	43	8083	7987	5203	2785
*41*	Guyana	Latin America & Caribbean	Upper middle income	6610	2019–2020	5061	14	5047	5207	1263	3944
*42*	Suriname	Latin America & Caribbean	Upper middle income	6938	2018	5952	95	5857	5888	4527	1361
*Total*	-	-	-	-	-	471364	1036	470328	1653850	1098981	554869

### 2.4. Statistical analysis

We used IBM SPSS Statistics 28.0 and Python 3.10.2 for the analysis. Descriptive statistics were used to determine actual group frequencies, percentages, minimum, maximum, and range. Pearson chi-square tests were utilized to examine the distribution of independent features concerning the dependent variable (use of reusable menstrual materials). We then used logistic regression to examine the association between demographic and country-level factors and the use of reusable menstrual materials (Adjusted for age, education, marital status, wealth index quintile, and availability of private places for washing menstrual materials, region, and country’s economy to observe the combined effects on the use of reusable menstrual materials)–and reported adjusted odds ratios and their 95% confidence intervals. We employed linear regression to test the linearity of use of reusable menstrual materials prevalence on GDP per capita. All tests were two-sided, with statistically significant values less than 0.05 and a 95% confidence interval. We utilized StataMP 16, Python 3.10.2, and RStudio 3.6.1 to visualize our results.

### 2.5. Ethical considerations

Since the data for the present study was obtained from secondary sources, ethical approval was not necessary for this study. The survey protocol received approval from the technical committee of the Government of Bangladesh, led by the Bangladesh Bureau of Statistics (BBS). In addition, each respondent provided verbal consent before participating. The interviewers emphasized that participation in the survey was entirely voluntary and assured that the information would be kept confidential. Respondents were given the option to decline answering any or specific questions, and they had the freedom to end the interview whenever they wished.

## 3. Results

This study analyzed data from 38 LMICs, including a total of 1653850 samples from 42 recent nationally representative surveys, with 1098981 from urban areas and 554869 from rural areas. All surveys were conducted between 2017 and 2019 (see **Table 1**).

The prevalence rates for the overall use of reusable menstrual materials, as well as for rural and urban areas, were 12.1% (95% CI: 12.1–12.2), 23.9% (95% CI: 23.8–24.0), and 6.2% (6.2–6.2), respectively. Sao Tome and Principe had the highest prevalence of use of reusable menstrual materials at 97.2% (95% CI: 96.5–97.9), followed by Chad at 84.6% (95% CI: 84.1–85.2), Madagascar at 77.9% (95% CI: 77.1–78.6), and Malawi at 70.4% (95% CI: 69.7–71.1). Serbia had the lowest overall frequency at 0.5% (95% CI: 0.3–0.8). The prevalence of use of reusable menstrual materials in urban areas was highest in Sao Tome and Principe at 97.7% (95% CI: 96.8–98.4), followed by Madagascar at 59.6% (95% CI: 58.1–61.2), Chad at 59.2% (95% CI: 57.6–60.8), Guinea Bissau at 57.7% (95% CI: 52.8–62.5), Nepal at 56.8% (95% CI: 55.7–57.8), Malawi at 53.6% (95% CI: 52.1–55.2), Bangladesh at 52.2% (95% CI: 51.3–53.0), and The Gambia at 51.6% (95% CI: 50.6–52.7). The lowest prevalence rate in urban areas was recorded in Serbia at 0.6% (95% CI: 0.3–1.0). On the other hand, Sao Tome and Principe had the highest rural prevalence of 96.5% (95% CI: 95.0–97.6), followed by Chad at 91.4% (95% CI: 90.9–91.8) and Sierra Leone at 90.8 (95% CI: 90.1–91.5). Serbia (0.5%) and Turkmenistan (0.5%) had the lowest prevalence rates of use of reusable menstrual materials in rural areas (see **Table 2**).

**Table 2:** Prevalence of the use of reusable menstrual materials among surveys.

Surveys	Urban	Rural	Overall
Algeria	3.9 (3.6–4.2)	7.8 (7.3–8.3)	5.3 (5.0–5.5)
Bangladesh	52.2 (51.3–53.0)	73.8 (73.4–74.3)	68.7 (68.3–69.0)
Central African Republic	41.7 (39.8–43.6)	80.4 (79.2–81.6)	65.3 (64.1–66.4)
Chad	59.2 (57.6–60.8)	91.4 (90.9–91.8)	84.6 (84.1–85.2)
Costa Rica	1.9 (1.9–1.9)	2.1 (2.0–2.1)	1.9 (1.9–2.0)
Cuba	1.9 (1.6–2.3)	4.1 (3.4–4.9)	2.7 (2.3–3.1)
Dominican Republic	2.0 (1.8–2.2)	3.1 (2.7–3.7)	2.2 (2.0–2.5)
DR Congo	36.8 (35.7–37.8)	84.2 (83.3–85.0)	58.8 (58.0–59.6)
Ghana	7.6 (6.9–8.2)	18.5 (17.5–19.5)	12.8 (12.2–13.4)
Guinea Bissau	57.7 (52.8–62.5)	73.5 (69.1–77.5)	65.8 (62.5–69.0)
Guyana	2.5 (1.7–3.5)	1.9 (1.5–2.4)	2.0 (1.7–2.5)
Honduras	2.1 (1.8–2.5)	4.1 (3.7–4.5)	3.1 (2.9–3.4)
Iraq	8.8 (8.3–9.4)	18.2 (17.1–19.3)	11.6 (11.1–12.1)
Kiribati	11.0 (9.7–12.4)	24.2 (22.0–26.5)	16.4 (15.1–17.6)
Kosovo (Roma settlements)	7.2 (5.5–9.3)	8.0 (6.0–10.4)	7.7 (6.3–9.2)
Kosovo	2.0 (1.5–2.7)	4.1 (3.4–4.9)	3.2 (2.7–3.7)
Kyrgyz Republic	8.2 (7.1–9.5)	25.7 (24.1–27.3)	18.6 (17.6–19.7)
Lao PDR	1.9 (1.6–2.3)	4.5 (4.1–4.9)	3.5 (3.2–3.7)
Lesotho	2.6 (2.0–3.2)	12.6 (11.4–13.8)	7.7 (7.0–8.5)
Madagascar	59.6 (58.1–61.2)	85.3 (84.5–86.0)	77.9 (77.1–78.6)
Malawi	53.6 (52.1–55.2)	74.4 (73.7–75.1)	70.4 (69.7–71.0)
Mongolia	1.9 (1.6–2.3)	5.0 (4.2–6.0)	2.8 (2.5–3.2)
Montenegro	4.1 (3.1–5.3)	4.3 (2.9–6.1)	4.2 (3.3–5.1)
Nepal	56.8 (55.7–57.8)	76.9 (75.5–78.2)	62.8 (61.9–63.6)
Pakistan Punjab	35.7 (35.0–36.3)	61.9 (61.4–62.4)	51.6 (51.2–52.0)
Pakistan Sindh	35.2 (34.3–36.0)	74.7 (73.8–75.6)	51.2 (50.5–51.8)
Republic of North Macedonia (Roma Settlements)	1.2 (0.7–2.0)	2.2 (0.3–7.9)	1.3 (0.8–2.1)
Republic of North Macedonia	0.8 (0.5–1.4)	1.0 (0.5–1.8)	0.9 (0.6–1.3)
Samoa	25.9 (22.8–29.2)	17.9 (16.5–19.4)	19.6 (18.3–21.0)
Sao Tome and Principe	97.7 (96.8–98.4)	96.5 (95.0–97.6)	97.2 (96.5–97.9)
Serbia (Roma Settlements)	2.9 (2.0–4.1)	4.5 (2.9–6.7)	3.4 (2.6–4.4)
Serbia	0.6 (0.3–1.0)	0.5 (0.2–1.0)	0.5 (0.3–0.8)
Sierra Leone	48.9 (47.7–50.1)	90.8 (90.1–91.5)	69.6 (68.8–70.4)
State of Palestine	2.3 (1.9–2.7)	2.1 (1.4–3.0)	2.2 (1.9–2.6)
Suriname	2.9 (2.5–3.5)	6.7 (5.4–8.1)	3.8 (3.3–4.3)
The Gambia	51.6 (50.6–52.7)	79.2 (77.8–80.6)	59.3 (58.4–60.1)
Togo	40.2 (38.4–41.9)	79.2 (77.7–80.7)	59.4 (58.1–60.6)
Tonga	1.2 (0.5–2.5)	0.9 (0.5–1.4)	1.0 (0.6–1.5)
Tunisia	2.7 (2.2–3.2)	6.4 (5.3–7.7)	3.8 (3.3–4.4)
Turkmenistan	1.3 (0.8–1.8)	0.5 (0.3–0.9)	0.9 (0.6–1.2)
Tuvalu	13.6 (10.6–17.0)	29.7 (23.7–36.4)	18.4 (15.6–21.5)
Zimbabwe	11.5 (10.4–12.6)	29.5 (28.2–30.8)	22.1 (21.2–23.0)
Total	6.2 (6.2–6.2)	23.9 (23.8–24.0)	12.1 (12.1–12.2)

For both overall and urban areas, the categories with the highest prevalence of use of reusable menstrual material were those who were younger, had less education and were currently married. Never-married women and girls from rural areas had a higher prevalence. Those who were poor and lived in rural areas, as well as those who were rich and lived in urban areas, had the highest prevalence. The prevalence was also higher among women and girls who were from lower- and lower-middle-income countries, lived in South Asia, West and Central Africa, and Eastern, and Southern Africa. Furthermore, this trend was observed among women who lacked access to private washing facilities for their menstrual materials [See **Table 3 and S1–S3 Tables**].

**Table 3:** Prevalence of the use of reusable menstrual materials among features.

Features		Urban	Rural	Overall
**Age**	15–19	7.4 (7.2–7.5)	26.5 (26.2–26.7)	14.8 (14.7–14.9)
	20–24	6.4 (6.3–6.5)	24.8 (24.5–25.1)	12.2 (12.1–12.4)
	25–29	6.6 (5.8–6.7)	24.3 (24.0–24.6)	12.4 (12.2–12.5)
	30–34	5.9 (5.8–6.0)	23.3 (23.1–23.6)	11.5 (11.4–11.6)
	35–39	6.1 (6.0–6.2)	22.5 (22.3–22.8)	11.8 (11.7–12.0)
	40–44	6.3 (6.1–6.4)	22.8 (22.3–23.1)	11.9 (11.8–12.1)
	45–49	4.1 (4.0–4.2)	20.9 (22.4–21.3)	9.0 (8.8–9.1)
**Education**	Primary or none	13.9 (13.7–14.0)	37.8 (37.6–38.0)	26.4 (26.2–26.5)
	Secondary	5.3 (5.2–5.4)	17.0 (16.8–17.1)	9.0 (8.9–9.1)
	Higher	3.0 (3.0–3.1)	4.7 (4.5–4.8)	3.3 (3.3–3.3)
**Union status**	Currently married/in union	7.3 (7.2–7.4)	27.9 (27.8–28.1)	15.2 (15.1–15.3)
	Formerly married/in union	4.3 (4.2–4.4)	15.9 (15.6–16.2)	7.3 (7.2–7.4)
	Never in union	5.5 (5.5–5.6)	18.4 (18.2–18.6)	9.3 (9.2–9.3)
**Wealth index quintile**	Poorest	5.7 (5.2–5.8)	24.8 (24.6–25.0)	16.5 (16.4–16.7)
	Second	5.3 (5.2–5.4)	26.0 (25.8–26.2)	14.5 (14.3–14.6)
	Middle	6.0 (5.9–6.1)	24.4 (24.1–24.6)	12.7 (12.6–12.8)
	Fourth	6.8 (6.5–6.6)	20.5 (24.1–20.8)	10.4 (10.3–10.5)
	Richest	6.5 (6.5–6.6)	18.5 (18.1–18.9)	7.7 (7.7–7.8)
**Region**	South Asia	42.4 (42.0–42.8)	69.3 (69.0–69.6)	58.8 (58.5–59.0)
	East Asia and the Pacific	4.3 (4.0–4.6)	7.8 (7.4–8.2)	6.2 (5.9–6.4)
	Europe and Central Asia	2.8 (2.6–3.1)	8.0 (7.5–8.4)	5.2 (5.0–5.5)
	West and Central Africa	42.3 (41.8–42.7)	77.6 (77.2–78.0)	60.7 (60.4–61.0)
	Middle East and North Africa	5.0 (4.8–5.2)	9.8 (9.4–10.3)	6.6 (6.4–6.8)
	Eastern and Southern Africa	34.9 (34.1–35.7)	65.5 (65.0–66.0)	56.5 (56.1–57.0)
	Latin America and Caribbean	1.9 (1.9–1.9)	2.2 (2.1–2.2)	2.0 (1.9–2.0)
**Country’s economy**	Lower	48.3 (47.8–48.8)	83.3 (83.0–83.6)	69.6 (69.3–69.8)
	Lower middle	23.0 (22.8–23.3)	47.3 (47.1–47.6)	36.1 (35.9–36.3)
	Upper middle	2.0 (2.0–2.0)	2.4 (2.3–2.4)	2.1 (2.1–2.1)
**Availability of private place for washing**	Yes	6.0 (6.0–6.1)	23.3 (23.2–23.4)	11.8 (11.7–11.8)
	No	12.0 (11.7–12.4)	40.1 (39.4–40.7)	23.7 (23.3–24.1)
**Total**	-	6.2 (6.2–6.2)	23.9 (23.8–24.0)	12.1 (12.1–12.2)

The prevalence of reusable menstrual materials was significantly associated with all independent variables examined, regardless of region (all p < 0.001). Specifically, younger women, those with lower education levels, and individuals from poorer economic backgrounds exhibited significantly higher prevalence rates of reusable menstrual materials in rural areas compared to urban areas. Furthermore, it was found that women who were currently married and had access to private washing spaces demonstrated a significantly higher prevalence rate of utilizing reusable options. [See **S4 Table**].

**Fig 1:**
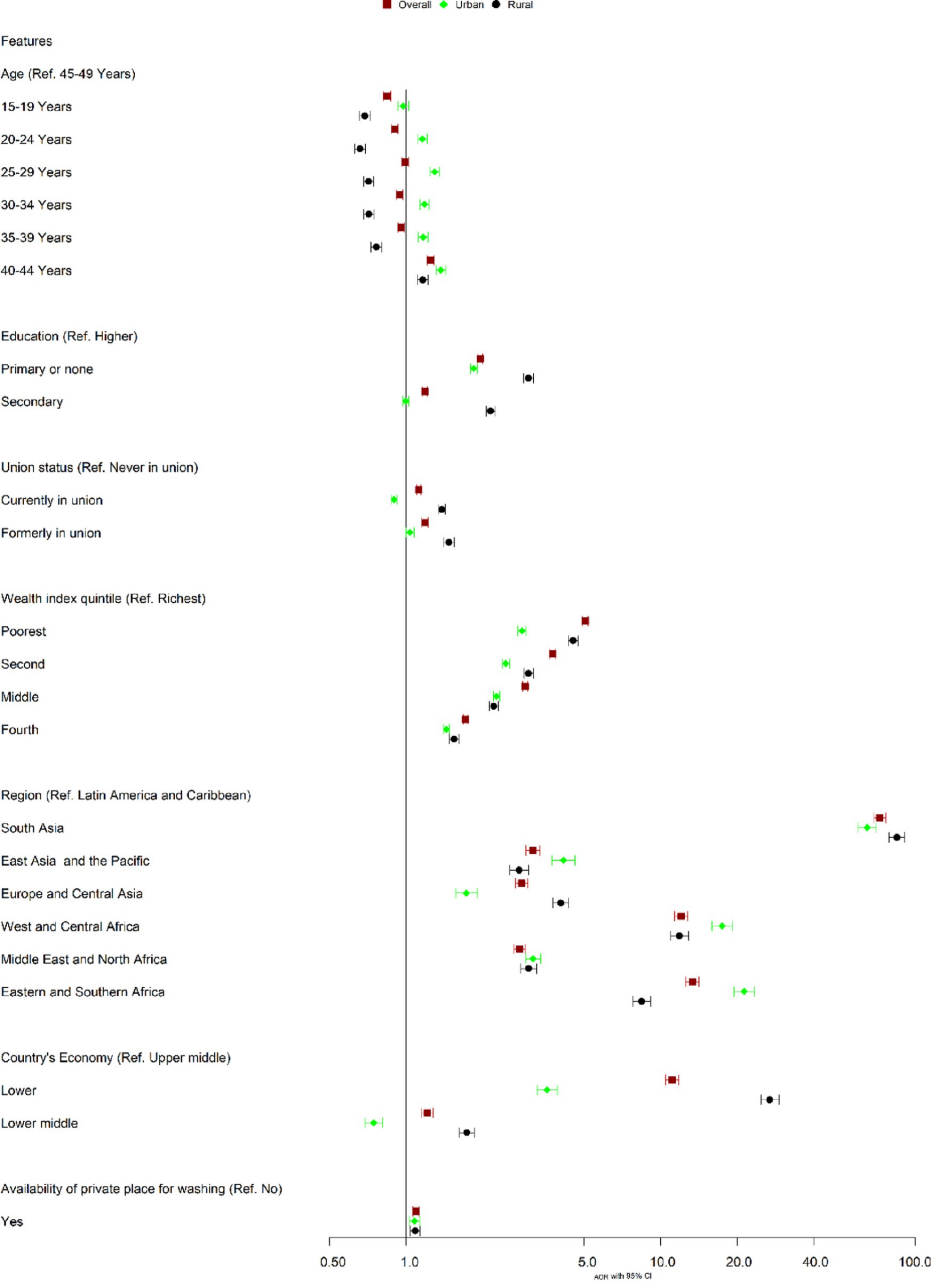
Adjusted odds ratios of the use of reusable menstrual materials among features by area (rural, urban, and overall). Note: Adjusted for age, education, marital status, wealth index quintile, and availability of private places for washing menstrual materials, region, and country’s economy.

Use of reusable menstrual materials was higher in women aged 40–44 years than in women aged 45–49 years in overall (AOR = 1.25, 95% CI: 1.21–1.29), rural (AOR = 1.16, 95% CI: 1.11–1.21), and urban (AOR = 1.37, 95% CI: 1.31–1.43) areas [See **Fig 1 and S5 Table**]. Women and girls with primary or no education were more likely than women and girls with higher education to use reusable menstrual materials in urban (AOR = 1.84, 95% CI: 1.79–1.90), rural (AOR = 3.03, 95% CI: 2.90–3.16), and overall areas (AOR = 1.96, 95% CI: 1.91–2.00). Furthermore, formerly married (divorced, separated, or the death of a spouse or partner) women and girls in rural areas (AOR = 1.47, 95% CI: 1.41–1.55), urban areas (AOR = 1.04, 95% CI: 1.0–1.07), and overall areas (AOR = 1.19, 95% CI: 1.15–1.22) were more likely to use of reusable menstrual materials than unmarried women and girls. In comparison to the richest, poor women and girls had higher use of reusable menstrual materials in urban (AOR = 2.86, 95%CI: 2.75–2.97), rural (AOR = 4.54, 95%CI: 4.34–4.74), and overall (AOR = 5.06, 95%CI: 4.94–5.18) locations.

South Asian women and girls were more likely to use reusable menstrual materials in urban (AOR = 64.94, 95% CI: 59.88–70.43), rural (AOR = 84.92, 95% CI: 79.18–91.08), and overall (AOR = 72.63, 95% CI: 68.91–76.54) areas, followed by Eastern and Southern Africa, West, and Central Africa, and Latin America and Caribbean. Low-income countries were more likely than upper-middle-income countries to use reusable menstrual materials in urban areas (AOR = 3.58, 95% CI: 3.27–3.93), rural areas (AOR = 26.90, 95% CI: 24.81–29.17), and overall areas (AOR = 11.10, 95% CI: 10.45–11.79). More crucially, women and girls who had a private washing place were more likely to use reusable menstrual materials in urban areas (AOR = 1.08, 95% CI: 1.03–1.13), rural areas (AOR = 1.09, 95% CI: 1.04–1.13), and overall areas (AOR = 1.09, 95% CI: 1.06–1.13) than those do not have private washing facility.

**Fig 2:**
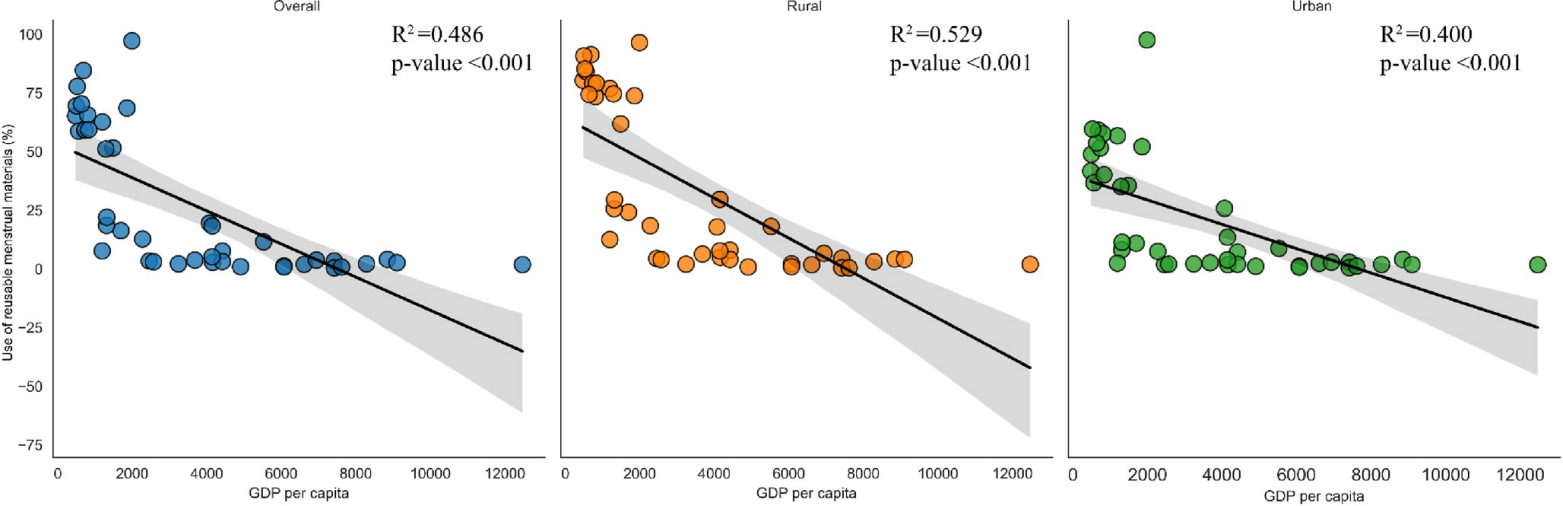
Linear regression of the use of reusable menstrual materials percentages according to their GDP per capita by area (rural, urban, and overall).

In the overall area’s linear regression model, the percentage of use of reusable menstrual materials decreases when the GDP per capita increases (β = -68.88, *p*-<0.001). Similar to the overall area, the prevalence of use of reusable menstrual materials decreases when GDP per capita increases both in rural (β = -61.97, *p*-<0.001<0.001), and urban areas (β = -77.06, *p*-<0.001) [see **Fig 2**].

## 4. Discussion

This study analyzed data from 42 nationally representative surveys in 38 LMICs to better understand the prevalence of the use of reusable menstrual material in both urban and rural areas. Out of a total of 1653850 samples, the pooled prevalence of the use of reusable menstrual materials was 12.1%. In this study, the prevalence of using reusable menstruation materials varied greatly by country, ranging from 0.5% in Serbia to 97.2% in Sao Tome and Principe. A study conducted in Nepal demonstrated that a significant proportion of women, specifically 66.7% before the earthquake and 76.1% after the earthquake, utilized reusable sanitary cloth [23]. Our findings in Nepal support this, with a similar prevalence of 62.8%. In the Indian context, it is worth noting that the utilization of reusable menstrual materials among women of reproductive age varied between 42% to 51.2% according to multiple studies [16, 24, 25]. However varying evidence is presented around these prevalence estimates as another research study conducted in Kolkata, India revealed that a notable proportion of women in the reproductive age group, specifically 6.5%, opt for the utilization of reusable menstrual materials [26]. However, because our analysis did not include India, these findings add context but are not directly comparable to our dataset. Similarly, studies from Ethiopia and Benin found lower prevalence rates among high school females (4.8% and 1.4%, respectively), but our study, which focused on MICS surveys, excluded data from these countries. The variations observed in these results can be attributed to disparities in the contextual and socio-demographic variables, the accessibility and affordability of menstrual products, and the influence of cultural norms and beliefs pertaining to menstrual materials [27, 28].

Interestingly, the prevalence of the use of reusable menstrual materials was much higher in rural areas, at 23.9%, compared to urban areas, where it was only 6.2%. The authors suggest that this may be due to a lack of access to single-use products, which are more commonly used in urban areas. However, this should not be taken as an indication that reusable products are inherently inferior; rather, it highlights the differences in accessibility and choice due to economic and geographic factors. Reusable cloth pads are often used in rural areas, largely due to their affordability, as many women and girls face financial barriers to purchasing single-use products [29]. Additionally, lower levels of education in rural areas may limit awareness about the range of available menstrual products and their proper usage [12]. This study highlights the importance of increasing access to a variety of menstrual products including both affordable, single-use options and high-quality reusable materials, especially in rural areas, as well as to provide education on menstrual hygiene management to empower informed choices.

Furthermore, the findings of this study revealed that women who were currently married or in committed relationships were more likely to use reusable materials compared to those who had never been married or in a committed relationship. This aligns with existing research, which indicates that never-married women often include a higher proportion of adolescents or young women, whereas married women are more representative of the adult female population. Previous research has shown that adult females tend to prefer reusable menstrual materials over disposable pads [30].

Moreover, our research also identified a significant difference in the usage of reusable menstrual materials based on the level of education. Specifically, use of reusable menstrual products was higher in women with lower educational attainment. This aligns with the expected trend, as women with higher education often have better information and knowledge about various menstrual products, have greater employment opportunities and purchasing power, enabling them to afford disposable sanitary pads. Women with a lower level of education in LMICs may have limited financial resources, and reusable options may be the more economical option over time. Nevertheless, it is crucial to acknowledge that reusable products are frequently selected for their long-term cost-effectiveness and environmental benefits, which are significant factors for many women, irrespective of their educational background. Consequently, it is crucial to disseminate comprehensive reproductive health education that enables women to make informed decisions based on their individual circumstances and preferences.

Notably, the utilization of reusable menstrual materials was observed to be more prevalent among women from lower socioeconomic strata. This trend may be influenced by financial constraints, which can limit access to commercially available sanitary pads. However, the decision to utilize reusable products should not be perceived as inferior or problematic; rather, it is a reflection of personal preference and economic realities. In LMICs, particularly in rural regions, access to a variety of menstruation products, including disposables, can be limited. Due to a lack of nearby stores or pricing concerns, women from lower socioeconomic backgrounds may have restricted access to disposable pads. Previous research in Ghana has highlighted that financial limitations represent a significant obstacle for adolescent girls in obtaining disposable sanitary products while attending school [31, 32]. This emphasizes the need of addressing disparities in menstrual hygiene management, particularly in low-income settings, where limited access to affordable and safe menstrual products can affect the health and well-being of women and girls.

Interestingly, reusable menstrual products were found to be most commonly used by women from both low-income rural areas and high-income urban areas. In rural areas, lower-income women may face challenges in accessing disposable menstrual products. As a result, they often turn to reusable options as a more affordable and accessible alternative. On the other hand, in wealthier urban areas, the widespread use of reusable products may be attributed to a greater understanding of menstrual health, a focus on environmental sustainability, and the easy access to high-quality reusable products. Members of this group may also be more informed about the advantages of utilizing reusable materials, such as long-term cost-effectiveness and decreased environmental impact. These contrasting patterns highlight the complexity of menstrual product use and underscore the importance of tailoring educational and policy interventions to address the specific needs and circumstances of different socioeconomic groups.

Access to safe and affordable menstrual products is crucial for menstruating individuals, but unfortunately, many worldwide lack the means to obtain them reliably. Reusable menstrual products, such as menstrual underwear, cloth pads, and menstrual cups, can cost between $15 and $40 per pair [33], $9 to $40 per napkin [18], and $25 or more per cup [34], respectively, depending on their location. While the initial cost may be high, reusable products can be used for two to six years for menstrual underwear and five to ten years for cloth pads, which significantly lowers the cost per menstrual cycle compared to disposable products. However, challenges with washing, drying, and changing reusable products have been reported frequently [18, 35, 36], and carrying used products and washing away menstrual blood can be seen as unpleasant and require privacy, time, water, soap, and equipment [18]. These necessities are often lacking in LMICs compared to high-income countries, where washing machines are prevalent [18]. Moreover, improper handling of reusable products can lead to infections. Therefore, it is essential to ensure that menstruating individuals have access to clean water and sanitation facilities to stay clean, comfortable, confident, informed, and free of infection, particularly in developing countries where access to these necessities is limited.

Disposing of menstrual waste can be a challenge for users, sanitation infrastructure, and the environment, with both safe and unsafe methods being used. Some commonly used methods, such as pit latrines, toilets, garbage, incineration/open burning, burying, and open dumping into ponds and fields, are rudimentary, unregulated, and potentially harmful. However, they may be the only available option for some users [37–39]. Non-biodegradable materials used in disposable sanitary pads can also be detrimental to the environment [37]. Studies in France, India, and the United States have shown that disposable pads score higher in terms of negative environmental effects than reusable pads [40]. Tampons, which contain at least 6% plastic, and menstrual pads, which contain 90% plastic, contribute to air pollution and global warming due to the large volumes of greenhouse gases released during their production [41]. Therefore, setting performance and quality criteria for reusable and biodegradable menstrual products is crucial for both women’s and girls’ health and waste reduction, as well as the environment. Safer and environmentally friendly options such as bamboo, banana, and water hyacinth fibers should be made available to women and girls at an affordable cost [12]. However, norms and regulations for menstrual products are lacking in several countries, making it essential to conduct performance research to establish high-quality standards for these products. This will ensure safe use and have significant implications for disposal and waste handling [42].

The use of reusable menstrual materials varies across regions, with South Asian women and girls using them the most, followed by those in Eastern and Southern Africa. In Central African countries, the Caribbean, and Latin America, reuse is not as widespread due to inconsistent access to menstrual materials and inadequate cleaning options for reusable menstrual products [43]. Management of menstrual products is a critical global issue beyond the physical and psychosocial impact that menstrual waste can have on young girls. Challenges in menstrual waste management, access to reusable materials [44], shame and stigma around discussing menstruation, and a lack of private cleaning and care spaces for women and girls make menstrual health a public and human rights issue globally. To promote healthy and dignified living for girls and women, men and boys must be included in discussions to break barriers and taboos around menstruation. Education and awareness on financial planning for menstrual products or safe ways to reuse materials through routine cleaning with soap and water can help address these issues [45].

It is important to acknowledge and address the inherent limitations present in our study. Initially, it is important to note that this study represents the most extensive investigation undertaken thus far on this specific topic. Furthermore, it is important to note that the utilization of MICS data limited our ability to conduct subnational analyses. This restriction may have obscured potential disparities in the use of reusable menstrual materials prevalence at the district level. The data utilized does not distinguish between reusable cloths and dirty rags, nor does it indicate respondents’ preferences for various products. Moreover, certain variables present in their questionnaire were regrettably absent from the dataset provided for our analysis. As a consequence, specific variables that could potentially influence the use of reusable menstrual materials were not taken into account. The overall prevalence estimate, fails to consider the differential population sizes across countries. Instead, these estimates assume an equal population size for each country. In conclusion, it is important to note that our binary-coded functional difficulties variable solely encompasses the challenges that have been incorporated into the dataset. However, it is crucial to acknowledge that this may not encompass the entirety of the use of all reusable menstrual materials (for example, menstrual cups).

## 5. Conclusions

The findings of this study highlight the widespread use of reusable menstrual materials in LMICs, specifically among women from lower socioeconomic status, with limited education, and living in rural regions. The practice is more prevalent in countries with lower GDP per capita, indicating a potential link to economic development. To address the widespread use of reusable menstrual materials among vulnerable populations in LMICs, policymakers should prioritize investments in sanitation infrastructure, particularly private washing spaces. In addition, it is crucial to promote reusable products through subsidies, awareness campaigns, and local production support. This study highlights the importance of implementing comprehensive educational interventions for menstrual health, which involve enhancing access to affordable menstrual products, improving sanitation facilities, and educating on menstrual hygiene management. It is essential to consider continuous research and data collection on the long-term impacts of both reusable and disposable menstrual products when making policy decisions. Future studies should aim to incorporate subnational data, examining specific preferences for menstrual products, and exploring the larger socio-cultural aspects that influence menstrual health management in diverse settings.

## Supporting information

S1 Table Bivariate association of features with the use of reusable menstrual materials (urban). (DOCX)

S2 Table Bivariate association of features with the use of reusable menstrual materials (rural). (DOCX)

S3 Table Bivariate association of features with the use of reusable menstrual materials (overall). (DOCX)

S4 Table Rural and urban differences in the prevalence of use of reusable menstrual materials. (DOCX)

S5 Table Adjusted odds ratio of use of reusable menstrual materials among features by area (urban, rural, overall). (DOCX)

S1 Dataset Dataset for the present study. (XLSX)
