# Supplementary material for: Cross-national variation in the prevalence and correlates of current use of reusable menstrual materials: Analysis of 42 cross-sectional surveys in low-income, lower-middle-income, and upper-middle-income countries
Source: PLoS One. 2024 Oct 7;19(10):e0310451. doi: 10.1371/journal.pone.0310451 (PMC11458041; doi:10.1371/journal.pone.0310451)
Supplement: S1 Table — (DOCX) [file pone.0310451.s001.docx]

**Supplement 1.** Bivariate association of features with the use of reusable menstrual materials (urban)

|  |  |  |  |  | |  |  |  |
| --- | --- | --- | --- | --- | --- | --- | --- | --- |
| **Features** | **Sub features** | **Total N = 1098981** | | **Menstrual materials reuse** | | | | ***p*-value** |
|  |  |  |  | **Yes** | | **No** | |  |
|  |  | **n** | **%** | **n** | **%** | **n** | **%** |  |
| **Age** | 15-19 | 157554 | 14.3 | 11620 | 7.4 | 145934 | 92.6 | <0.001 |
|  | 20-24 | 189154 | 17.2 | 12117 | 6.4 | 177037 | 93.6 |  |
|  | 25-29 | 180508 | 16.4 | 11972 | 6.6 | 168536 | 93.4 |  |
|  | 30-34 | 177243 | 16.1 | 10425 | 5.9 | 166818 | 94.1 |  |
|  | 35-39 | 152350 | 13.9 | 9265 | 6.1 | 143085 | 93.9 |  |
|  | 40-44 | 128701 | 11.7 | 8054 | 6.3 | 120647 | 93.7 |  |
|  | 45-49 | 113471 | 10.3 | 4665 | 4.1 | 108806 | 95.9 |  |
| **Education** | Primary or none | 215063 | 19.6 | 29871 | 13.9 | 185192 | 86.1 | <0.001 |
|  | Secondary | 501376 | 45.6 | 26623 | 5.3 | 474753 | 94.7 |  |
|  | Higher | 382542 | 34.8 | 11624 | 3.0 | 370918 | 97.0 |  |
| **Union status** | Currently married/in union | 541070 | 49.2 | 39419 | 7.3 | 501651 | 92.7 | <0.001 |
|  | Formerly married/in union | 175016 | 15.9 | 7459 | 4.3 | 167557 | 95.7 |  |
|  | Never in union | 382895 | 34.8 | 21241 | 5.5 | 361654 | 94.5 |  |
| **Wealth index quintile** | Poorest | 122212 | 11.1 | 6930 | 5.7 | 115282 | 94.3 | <0.001 |
|  | Second | 178827 | 16.3 | 9474 | 5.3 | 169353 | 94.7 |  |
|  | Middle | 219779 | 20.0 | 13188 | 6.0 | 206591 | 94.0 |  |
|  | Fourth | 253993 | 23.1 | 17340 | 6.8 | 236653 | 93.2 |  |
|  | Richest | 324171 | 29.5 | 21187 | 6.5 | 302984 | 93.5 |  |
| **Region** | South Asia | 59250 | 5.4 | 25148 | 42.4 | 34102 | 57.6 | <0.001 |
|  | East Asia and the Pacific | 17276 | 1.6 | 746 | 4.3 | 16530 | 95.7 |  |
|  | Europe and Central Asia | 14952 | 1.4 | 425 | 2.8 | 14527 | 97.2 |  |
|  | West and Central Africa | 41670 | 3.8 | 17615 | 42.3 | 24055 | 57.7 |  |
|  | Middle East and North Africa | 39932 | 3.6 | 1986 | 5.0 | 37946 | 95.0 |  |
|  | Eastern and Southern Africa | 13852 | 1.3 | 4836 | 34.9 | 9016 | 65.1 |  |
|  | Latin America and Caribbean | 912049 | 83.0 | 17363 | 1.9 | 894686 | 98.1 |  |
| **Country's economy** | Lower | 41299 | 3.8 | 19947 | 48.3 | 21352 | 51.7 | <0.001 |
|  | Lower middle | 128812 | 11.7 | 29655 | 23.0 | 99157 | 77.0 |  |
|  | Upper middle | 928869 | 84.5 | 18516 | 2.0 | 910353 | 98.0 |  |
| **Availability of private place for washing** | Yes | 1070702 | 97.4 | 64712 | 6.0 | 1005990 | 94.0 | <0.001 |
|  | No | 28279 | 2.6 | 3406 | 12.0 | 24873 | 88.0 |  |
| **Total** |  | 1098981 | 100.0 | 68118 | 6.2 | 1030863 | 93.8 |  |
