# Supplementary material for: Cross-national variation in the prevalence and correlates of current use of reusable menstrual materials: Analysis of 42 cross-sectional surveys in low-income, lower-middle-income, and upper-middle-income countries
Source: PLoS One. 2024 Oct 7;19(10):e0310451. doi: 10.1371/journal.pone.0310451 (PMC11458041; doi:10.1371/journal.pone.0310451)
Supplement: S2 Table — (DOCX) [file pone.0310451.s002.docx]

**Supplement 2.** Bivariate association of features with the use of reusable menstrual materials (rural)

|  |  |  |  |  |  |  |  |  |
| --- | --- | --- | --- | --- | --- | --- | --- | --- |
| **Features** | **Sub features** | **Total N = 554869** | | **Menstrual materials reuse** | | | | ***p*-value** |
|  |  |  |  | **Yes** | | **No** | |  |
|  |  | **n** | **%** | **n** | **%** | **n** | **%** |  |
| **Age** | 15-19 | 100333 | 18.1 | 26545 | 26.5 | 73788 | 73.5 | <0.001 |
|  | 20-24 | 88032 | 15.9 | 21809 | 24.8 | 66223 | 75.2 |  |
|  | 25-29 | 86755 | 15.6 | 21070 | 24.3 | 65685 | 75.7 |  |
|  | 30-34 | 84306 | 15.2 | 19683 | 23.3 | 64623 | 76.7 |  |
|  | 35-39 | 81814 | 14.7 | 18447 | 22.5 | 63367 | 77.5 |  |
|  | 40-44 | 67568 | 12.2 | 15380 | 22.8 | 52188 | 77.2 |  |
|  | 45-49 | 46060 | 8.3 | 9646 | 20.9 | 36414 | 79.1 |  |
| **Education** | Primary or none | 235199 | 42.4 | 88902 | 37.8 | 146297 | 62.2 | <0.001 |
|  | Secondary | 233913 | 42.2 | 39658 | 17.0 | 194255 | 83.0 |  |
|  | Higher | 85757 | 15.5 | 4020 | 4.7 | 81737 | 95.3 |  |
| **Union status** | Currently married/in union | 337238 | 60.8 | 94106 | 27.9 | 243132 | 72.1 | <0.001 |
|  | Formerly married/in union | 62526 | 11.3 | 9942 | 15.9 | 52584 | 84.1 |  |
|  | Never in union | 155104 | 28.0 | 28532 | 18.4 | 126572 | 81.6 |  |
| **Wealth index quintile** | Poorest | 161164 | 29.0 | 39933 | 24.8 | 121231 | 75.2 | <0.001 |
|  | Second | 141773 | 25.6 | 36859 | 26.0 | 104914 | 74.0 |  |
|  | Middle | 125209 | 22.6 | 30533 | 24.4 | 94676 | 75.6 |  |
|  | Fourth | 90206 | 16.3 | 18496 | 20.5 | 71710 | 79.5 |  |
|  | Richest | 36516 | 6.6 | 6759 | 18.5 | 29757 | 81.5 |  |
| **Region** | South Asia | 92466 | 16.7 | 64036 | 69.3 | 28430 | 30.7 | <0.001 |
|  | East Asia and the Pacific | 19752 | 3.6 | 1536 | 7.8 | 18216 | 92.2 |  |
|  | Europe and Central Asia | 12807 | 2.3 | 1020 | 8.0 | 11787 | 92.0 |  |
|  | West and Central Africa | 45350 | 8.2 | 35202 | 77.6 | 10148 | 22.4 |  |
|  | Middle East and North Africa | 19235 | 3.5 | 1891 | 9.8 | 17344 | 90.2 |  |
|  | Eastern and Southern Africa | 33161 | 6.0 | 21731 | 65.5 | 11430 | 34.5 |  |
|  | Latin America and Caribbean | 332097 | 59.9 | 7164 | 2.2 | 324933 | 97.8 |  |
| **Country's economy** | Lower | 63836 | 11.5 | 53188 | 83.3 | 10648 | 16.7 | <0.001 |
|  | Lower middle | 150874 | 27.2 | 71380 | 47.3 | 79494 | 52.7 |  |
|  | Upper middle | 340158 | 61.3 | 8012 | 2.4 | 332146 | 97.6 |  |
| **Availability of private place for washing** | Yes | 534795 | 96.4 | 124538 | 23.3 | 410257 | 76.7 | <0.001 |
|  | No | 20074 | 3.6 | 8042 | 40.1 | 12032 | 59.9 |  |
| **Total** |  | 554869 | 100.0 | 132580 | 23.9 | 422289 | 76.1 |  |
