# Supplementary material for: Cross-national variation in the prevalence and correlates of current use of reusable menstrual materials: Analysis of 42 cross-sectional surveys in low-income, lower-middle-income, and upper-middle-income countries
Source: PLoS One. 2024 Oct 7;19(10):e0310451. doi: 10.1371/journal.pone.0310451 (PMC11458041; doi:10.1371/journal.pone.0310451)
Supplement: S3 Table — (DOCX) [file pone.0310451.s003.docx]

**Supplement 3.** Bivariate association of features with the use of reusable menstrual materials (overall)

|  |  |  |  |  |  |  |  |  |
| --- | --- | --- | --- | --- | --- | --- | --- | --- |
| **Features** | **Sub features** | **Total N = 1653850** | | **Menstrual materials reuse** | | | | ***p*-value** |
|  |  |  |  | **Yes** | | **No** | |  |
|  |  | **n** | **%** | **n** | **%** | **n** | **%** |  |
| **Age** | 15-19 | 257886 | 15.6 | 38164 | 14.8 | 219722 | 85.2 | <0.001 |
|  | 20-24 | 277186 | 16.8 | 33926 | 12.2 | 243260 | 87.8 |  |
|  | 25-29 | 267263 | 16.2 | 33042 | 12.4 | 234221 | 87.6 |  |
|  | 30-34 | 261549 | 15.8 | 30108 | 11.5 | 231441 | 88.5 |  |
|  | 35-39 | 234165 | 14.2 | 27713 | 11.8 | 206452 | 88.2 |  |
|  | 40-44 | 196270 | 11.9 | 23435 | 11.9 | 172835 | 88.1 |  |
|  | 45-49 | 159531 | 9.6 | 14311 | 9.0 | 145220 | 91.0 |  |
| **Education** | Primary or none | 450262 | 27.2 | 118773 | 26.4 | 331489 | 73.6 | <0.001 |
|  | Secondary | 735289 | 44.5 | 66281 | 9.0 | 669008 | 91.0 |  |
|  | Higher | 468299 | 28.3 | 15644 | 3.3 | 452655 | 96.7 |  |
| **Union status** | Currently married/in union | 878309 | 53.1 | 133525 | 15.2 | 744784 | 84.8 | <0.001 |
|  | Formerly married/in union | 237542 | 14.4 | 17401 | 7.3 | 220141 | 92.7 |  |
|  | Never in union | 537998 | 32.5 | 49772 | 9.3 | 488226 | 90.7 |  |
| **Wealth index quintile** | Poorest | 283377 | 17.1 | 46863 | 16.5 | 236514 | 83.5 | <0.001 |
|  | Second | 320600 | 19.4 | 46333 | 14.5 | 274267 | 85.5 |  |
|  | Middle | 344987 | 20.9 | 43721 | 12.7 | 301266 | 87.3 |  |
|  | Fourth | 344198 | 20.8 | 35835 | 10.4 | 308363 | 89.6 |  |
|  | Richest | 360687 | 21.8 | 27946 | 7.7 | 332741 | 92.3 |  |
| **Region** | South Asia | 151716 | 9.2 | 89184 | 58.8 | 62532 | 41.2 | <0.001 |
|  | East Asia and the Pacific | 37028 | 2.2 | 2282 | 6.2 | 34746 | 93.8 |  |
|  | Europe and Central Asia | 27760 | 1.7 | 1446 | 5.2 | 26314 | 94.8 |  |
|  | West and Central Africa | 87020 | 5.3 | 52817 | 60.7 | 34203 | 39.3 |  |
|  | Middle East and North Africa | 59168 | 3.6 | 3877 | 6.6 | 55291 | 93.4 |  |
|  | Eastern and Southern Africa | 47013 | 2.8 | 26567 | 56.5 | 20446 | 43.5 |  |
|  | Latin America and Caribbean | 1244146 | 75.2 | 24526 | 2.0 | 1219620 | 98.0 |  |
| **Country's economy** | Lower | 105135 | 6.4 | 73135 | 69.6 | 32000 | 30.4 | <0.001 |
|  | Lower middle | 279687 | 16.9 | 101035 | 36.1 | 178652 | 63.9 |  |
|  | Upper middle | 1269027 | 76.7 | 26528 | 2.1 | 1242499 | 97.9 |  |
| **Availability of private place for washing** | Yes | 1605497 | 97.1 | 189250 | 11.8 | 1416247 | 88.2 | <0.001 |
|  | No | 48352 | 2.9 | 11448 | 23.7 | 36904 | 76.3 |  |
| **Total** |  | 1653850 | 100.0 | 200699 | 12.1 | 1453151 | 87.9 |  |
