# Supplementary material for: Cross-national variation in the prevalence and correlates of current use of reusable menstrual materials: Analysis of 42 cross-sectional surveys in low-income, lower-middle-income, and upper-middle-income countries
Source: PLoS One. 2024 Oct 7;19(10):e0310451. doi: 10.1371/journal.pone.0310451 (PMC11458041; doi:10.1371/journal.pone.0310451)
Supplement: S4 Table — (DOCX) [file pone.0310451.s004.docx]

**Supplement 4:** Rural and urban differences in the prevalence of use of reusable menstrual materials

|  |  |  | |  | |  |
| --- | --- | --- | --- | --- | --- | --- |
| **Features** | **Sub features** | **Menstrual materials reuse** | | **Menstrual materials reuse** | | ***p*-value** |
|  |  | **Yes (urban)** | | **Yes (rural)** | |  |
|  |  | **n** | **%** | **n** | **%** |  |
| **Age** | 15-19 | 11620 | 7.4 | 26545 | 26.5 | <0.001 |
|  | 20-24 | 12117 | 6.4 | 21809 | 24.8 | <0.001 |
|  | 25-29 | 11972 | 6.6 | 21070 | 24.3 | <0.001 |
|  | 30-34 | 10425 | 5.9 | 19683 | 23.3 | <0.001 |
|  | 35-39 | 9265 | 6.1 | 18447 | 22.5 | <0.001 |
|  | 40-44 | 8054 | 6.3 | 15380 | 22.8 | <0.001 |
|  | 45-49 | 4665 | 4.1 | 9646 | 20.9 | <0.001 |
| **Education** | Primary or none | 29871 | 13.9 | 88902 | 37.8 | <0.001 |
|  | Secondary | 26623 | 5.3 | 39658 | 17.0 | <0.001 |
|  | Higher | 11624 | 3.0 | 4020 | 4.7 | <0.001 |
| **Union status** | Currently married/in union | 39419 | 7.3 | 94106 | 27.9 | <0.001 |
|  | Formerly married/in union | 7459 | 4.3 | 9942 | 15.9 | <0.001 |
|  | Never in union | 21241 | 5.5 | 28532 | 18.4 | <0.001 |
| **Wealth index quintile** | Poorest | 6930 | 5.7 | 39933 | 24.8 | <0.001 |
|  | Second | 9474 | 5.3 | 36859 | 26.0 | <0.001 |
|  | Middle | 13188 | 6.0 | 30533 | 24.4 | <0.001 |
|  | Fourth | 17340 | 6.8 | 18496 | 20.5 | <0.001 |
|  | Richest | 21187 | 6.5 | 6759 | 18.5 | <0.001 |
| **Region** | South Asia | 25148 | 42.4 | 64036 | 69.3 | <0.001 |
|  | East Asia and the Pacific | 746 | 4.3 | 1536 | 7.8 | <0.001 |
|  | Europe and Central Asia | 425 | 2.8 | 1020 | 8.0 | <0.001 |
|  | West and Central Africa | 17615 | 42.3 | 35202 | 77.6 | <0.001 |
|  | Middle East and North Africa | 1986 | 5.0 | 1891 | 9.8 | <0.001 |
|  | Eastern and Southern Africa | 4836 | 34.9 | 21731 | 65.5 | <0.001 |
|  | Latin America and Caribbean | 17363 | 1.9 | 7164 | 2.2 | <0.001 |
| **Country's economy** | Lower | 19947 | 48.3 | 53188 | 83.3 | <0.001 |
|  | Lower middle | 29655 | 23 | 71380 | 47.3 | <0.001 |
|  | Upper middle | 18516 | 2.0 | 8012 | 2.4 | <0.001 |
| **Availability of private place for washing** | Yes | 64712 | 6.0 | 124538 | 23.3 | <0.001 |
|  | No | 3406 | 12.0 | 8042 | 40.1 | <0.001 |
| **Total** |  | 68118 | 6.2 | 132580 | 23.9 | <0.001 |
