# Supplementary material for: Cross-national variation in the prevalence and correlates of current use of reusable menstrual materials: Analysis of 42 cross-sectional surveys in low-income, lower-middle-income, and upper-middle-income countries
Source: PLoS One. 2024 Oct 7;19(10):e0310451. doi: 10.1371/journal.pone.0310451 (PMC11458041; doi:10.1371/journal.pone.0310451)
Supplement: S5 Table — (DOCX) [file pone.0310451.s005.docx]

**Supplement 5.** Adjusted odds ratio of use of reusable menstrual materials among features by area (Urban, Rural, Overall)

|  | **Urban** | | | | **Rural** | | | | **Overall** | | | |
| --- | --- | --- | --- | --- | --- | --- | --- | --- | --- | --- | --- | --- |
| **Features (Reference)** | ***p*-value** | **AOR** | **95% CI**  **LL UL** | | ***p*-value** | **AOR** | **95% CI**  **LL UL** | | ***p*-value** | **AOR** | **95% CI**  **LL UL** | |
| **Age (Ref. 45-49)** |  |  |  |  |  |  |  |  |  |  |  |  |
| 15-19 | 0.275 | 0.97 | 0.93 | 1.02 | <0.001 | 0.69 | 0.65 | 0.72 | <0.001 | 0.84 | 0.81 | 0.87 |
| 20-24 | <0.001 | 1.16 | 1.11 | 1.21 | <0.001 | 0.66 | 0.63 | 0.69 | <0.001 | 0.90 | 0.87 | 0.93 |
| 25-29 | <0.001 | 1.30 | 1.24 | 1.35 | <0.001 | 0.71 | 0.68 | 0.75 | 0.464 | 0.99 | 0.96 | 1.02 |
| 30-34 | <0.001 | 1.18 | 1.14 | 1.23 | <0.001 | 0.71 | 0.68 | 0.75 | <0.001 | 0.94 | 0.91 | 0.97 |
| 35-39 | <0.001 | 1.17 | 1.12 | 1.22 | <0.001 | 0.76 | 0.73 | 0.80 | 0.003 | 0.96 | 0.93 | 0.99 |
| 40-44 | <0.001 | 1.37 | 1.31 | 1.43 | <0.001 | 1.16 | 1.11 | 1.22 | <0.001 | 1.25 | 1.21 | 1.29 |
| **Education (Ref. Higher)** |  |  |  |  |  |  |  |  |  |  |  |  |
| Primary or none | <0.001 | 1.84 | 1.79 | 1.90 | <0.001 | 3.03 | 2.90 | 3.16 | <0.001 | 1.96 | 1.91 | 2.00 |
| Secondary | 0.834 | 1.00 | 0.97 | 1.02 | <0.001 | 2.15 | 2.06 | 2.24 | <0.001 | 1.19 | 1.16 | 1.21 |
| **Union status (Ref. Never in union)** |  |  |  |  |  |  |  |  |  |  |  |  |
| Currently in union | <0.001 | 0.90 | 0.87 | 0.92 | <0.001 | 1.38 | 1.34 | 1.43 | <0.001 | 1.12 | 1.10 | 1.14 |
| Formerly in union | 0.055 | 1.04 | 1.00 | 1.07 | <0.001 | 1.47 | 1.41 | 1.55 | <0.001 | 1.19 | 1.15 | 1.22 |
| **Wealth index quintile (Ref. Richest)** |  |  |  |  |  |  |  |  |  |  |  |  |
| Poorest | <0.001 | 2.86 | 2.75 | 2.97 | <0.001 | 4.54 | 4.34 | 4.74 | <0.001 | 5.06 | 4.94 | 5.18 |
| Second | <0.001 | 2.46 | 2.38 | 2.55 | <0.001 | 3.03 | 2.91 | 3.16 | <0.001 | 3.77 | 3.68 | 3.85 |
| Middle | <0.001 | 2.27 | 2.20 | 2.34 | <0.001 | 2.21 | 2.12 | 2.30 | <0.001 | 2.93 | 2.87 | 3.00 |
| Fourth | <0.001 | 1.44 | 1.40 | 1.48 | <0.001 | 1.55 | 1.48 | 1.61 | <0.001 | 1.71 | 1.67 | 1.75 |
| **Region (Ref. Latin America and Caribbean)** |  |  |  |  |  |  |  |  |  |  |  |  |
| South Asia | <0.001 | 64.94 | 59.88 | 70.43 | <0.001 | 84.92 | 79.18 | 91.08 | <0.001 | 72.63 | 68.91 | 76.54 |
| East Asia and the Pacific | <0.001 | 4.15 | 3.74 | 4.61 | <0.001 | 2.78 | 2.56 | 3.02 | <0.001 | 3.15 | 2.95 | 3.36 |
| Europe and Central Asia | <0.001 | 1.73 | 1.56 | 1.91 | <0.001 | 4.05 | 3.77 | 4.35 | <0.001 | 2.84 | 2.69 | 3.01 |
| West and Central Africa | <0.001 | 17.44 | 15.91 | 19.13 | <0.001 | 11.87 | 10.95 | 12.86 | <0.001 | 12.05 | 11.35 | 12.80 |
| Middle East and North Africa | <0.001 | 3.16 | 2.95 | 3.37 | <0.001 | 3.03 | 2.82 | 3.26 | <0.001 | 2.79 | 2.66 | 2.94 |
| Eastern and Southern Africa | <0.001 | 21.34 | 19.42 | 23.45 | <0.001 | 8.43 | 7.78 | 9.14 | <0.001 | 13.33 | 12.54 | 14.16 |
| **Country's Economy (Ref. Upper middle)** |  |  |  |  |  |  |  |  |  |  |  |  |
| Lower | <0.001 | 3.58 | 3.27 | 3.93 | <0.001 | 26.90 | 24.81 | 29.17 | <0.001 | 11.10 | 10.45 | 11.79 |
| Lower middle | <0.001 | 0.75 | 0.69 | 0.81 | <0.001 | 1.73 | 1.62 | 1.85 | <0.001 | 1.21 | 1.15 | 1.28 |
| **Availability of private place for washing (Ref. No)** |  |  |  |  |  |  |  |  |  |  |  |  |
| Yes | 0.001 | 1.08 | 1.03 | 1.13 | <0.001 | 1.09 | 1.04 | 1.13 | <0.001 | 1.09 | 1.06 | 1.13 |

**Note:** Adjusted for age, education, marital status, wealth index quintile, and availability of private places for washing menstrual materials, region, and country’s economy.
